# Retraction: Quality of Life and PTSD Symptoms, and Temperament and Coping With Stress

**DOI:** 10.3389/fpsyg.2020.01174

**Published:** 2020-05-12

**Authors:** Frontiers Editorial Office

Following publication, concerns were raised about the dual publication of this article in Frontiers in Psychology and Current Psychology. An investigation was conducted in accordance with our established procedures, whereby it was confirmed that the authors submitted the manuscript for consideration in two journals simultaneously. Following the Committee on Publication Ethics guidelines, this article, published most recently will be retracted.

This retraction was approved by the Chief Editors of Frontiers in Psychology and the Editor-in-Chief of Frontiers. The authors agree to this retraction and apologize to the readers and Editors.

